# The impact of preoperative etiology on emergent pancreaticoduodenectomy for non-traumatic patients

**DOI:** 10.1186/s13017-017-0133-6

**Published:** 2017-05-02

**Authors:** Chun-Yi Tsai, Bo-Ru Lai, Shang-Yu Wang, Chien-Hung Liao, Yu-Yin Liu, Shih-Ching Kang, Chun-Nan Yeh, Yi-Yin Jan, Ta-Sen Yeh

**Affiliations:** 1 Department of General Surgery, Chang Gung Memorial Hospital, Chang Gung University, Linkou branch, No.5, Fu-Xing Street, Kueishan District, Taoyuan City, 333 Taiwan; 2 Chang Gung University, No. 259, Wen Hua First Road, Kueishan District, Taoyuan City, 333 Taiwan; 3 Department of Traumatology and Emergent Surgery, Chang Gung Memorial Hospital, Chang Gung University, Linkou branch, No.5, Fu-Xing Street, Kueishan District, Taoyuan City, 333 Taiwan

**Keywords:** Pancreaticoduodenectomy, Emergent pancreaticoduodenectomy, Gastrointestinal bleeding, Gastrointestinal perforation, Surgical complication, Lethality

## Abstract

**Background:**

Emergent pancreaticoduodenectomy is a life-saving procedure in certain clinical scenarios when all the conservative treatment fails. The indications can be limited into perforation and bleeding. To clarify the impact of etiology on surgical outcomes of emergent pancreaticoduodenectomy for non-trauma, we analyzed our patients and performed a literature review.

**Methods:**

We reviewed 931 consecutive pancreaticoduodenectomies performed at our institute between January 2001 and July 2015. Patients with emergent pancreaticoduodenectomy for non-trauma etiologies were enrolled, whereas those who suffered from caustic injuries were excluded. The keywords “emergent/emergency” and “pancreaticoduodenectomy/pancreatoduodenectomy” were applied in a literature search. The universally available data for all the enrolled patients including etiology, surgical complications, outcomes, and hospital stays were analyzed. Univariate and multivariate logistic analysis for the contributing factors to surgical mortality were performed.

**Results:**

Six out of 931 (0.6%) registered pancreaticoduodenectomies matched our criteria of inclusion. The literature review obtained 4 series and 7 case reports, which when combined with our patients yielded a cohort of 31 emergent pancreaticoduodenectomies with 13 cases of perforation and 18 of bleeding. The rate of emergent pancreaticoduodenectomy for non-traumatic etiologies is similar between the present study and the other 3 series, ranging from 0.3 to 3%. The overall surgical complication rate was 83.9%. The rate of surgical mortality is significantly higher than in elective pancreaticoduodenectomy by propensity score matching with age and gender (19.4 versus 3.2%, *P* = 0.015). Univariate and multivariate logistic regression disclosed that etiology is the only preoperative risk factor for surgical mortality (perforation versus bleeding; odds ratio = 39.494, *P* = 0.031).

**Conclusions:**

Emergent pancreaticoduodenectomy remains a rare operation. Surgical morbidity and mortality are higher than with elective pancreaticoduodenectomy among different reported series. By sorting the preoperative etiologies into two groups, perforation carries a higher risk of surgical mortality than bleeding.

## Background

Pancreaticoduodenectomy (PD) is a procedure for managing lesions of various etiologies involving the region of duodenum, distal bile duct, and pancreatic head [1, 2]. Due to the refinement of preoperative management and surgical techniques, surgical morbidity and mortality rates of PD have decreased in high-volume institutes in recent decades [3, 4]. However, it is still a technically demanding and complex procedure. Regardless of the prognosis of underlying malignancies, which are the majority of indications for patients undergoing PD, the complications of PD have been meticulously studied [5]. Emergent PD (EPD) were encountered by surgeons in traumatic scenarios [6–9]. EPD for non-traumatic etiologies, such as perforation-related peritonitis or uncontrolled bleeding around the pancreaticoduodenal area, has been sporadically reported. In order to minimize the complications caused by pancreaticojejunostomy, some authors proposed a “staged reconstruction” and obtained satisfying results [10]. Sakakima et al [11] reported a successful EPD with a similar concept of staged reconstruction of pancreaticojejunostomy. In contrast to reports focusing on operative management, there have been no reports regarding preoperative predictors for surgical outcomes of EPDs. As one of the referring centers in Taiwan, we reviewed our patients and cases from the literature that underwent EPD for non-traumatic etiologies to clarify the impact of etiology on the outcomes of EPD.

## Methods

Between January 2000 and July 2015, 931 consecutive PDs (classic Whipple’s operation and pylorus-preserving PD) were performed at the Department of Surgery, Chang Gung Memorial Hospital, Linkou, Taiwan. The indications for emergent PD of non-traumatic etiologies include (1) incomplete preoperative preparation and management for neoplastic lesions (2) operation for failed non-surgical hemostasis (3) emergent operations for peritonitis/intra-abdominal infection/ or iatrogenic perforations. Patients necessitating EPDs due to caustic injuries were excluded because of multivisceral resection and the systemically catastrophic cascade resulting from digestive chemicals [12]. Patients who met the inclusion criteria were analyzed regarding gender, age, etiology, body mass index (BMI), underlying diseases, preoperative severity (APACHE II score), the interval to the reconstruction, the length of hospital stay, surgical complications, and mortality. Surgical complications were described and classified according to the Clavien-Dindo classification [13]. Surgical mortality is defined as death either within 30 days or during the same admission after EPD. For patients without surgical mortality, we reviewed their performance and sequelae during follow-up.

The literature review was performed via MEDLINE and PubMed focusing on English language literatures with the keywords, “emergent/emergency” and “pancreaticoduodenectomy/pancreatoduodenectomy”. We then selected only literatures with full text published between the year 2001 and 2015. From the series obtained by this combination, including case reports, the patients designated as non-traumatic etiologies were extracted and included in our study. Statistical analyses were done with IBM SPSS. Values of *P < 0.05* were considered statistically significant. All results were expressed as mean +/− standard deviations. Univariate and multivariate logistic regression analyses were applied to determine the factors affecting surgical mortality.

## Results

**Fig. 1:**
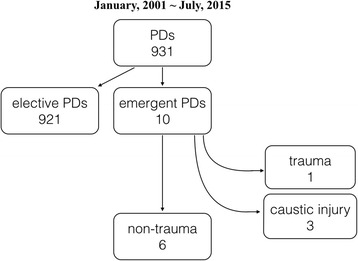
The flow chart of patient selection. *PD* pancreaticoduodenectomy

From a total of 931 patients with PD, only 6 underwent EPD from 2001 to 2015 (Table 1 and Fig. 1). There were 3 male and 3 female patients with mean age 53.5 years old (range: 32 to 78). The clinical scenarios of these patients were described as following: the first patient underwent partial pancreactomy near pancreatic head and complicated with hemorrhagic shock. He underwent EPD after failure of bleeding control by angiographic embolization. The second patient presented to emergency department with a massively bleeding duodenal tumor and failed to angiographic embolization. He underwent EPD to treat the life-threatening bleeding and finally diagnosed with lymphoma. The third patient had underlying gastric cancer status post subtotal gastrectomy with Billroth II gastrojejunostomy. She received endoscopic retrograde cholangiopancreatography (ERCP) for choledocholithotomy and complicated with afferent loop perforation. Owing to delayed diagnosis of perforation, the patient developed sepsis, and the regional bowel tissue disclosed insufficiency. EPD was performed by the surgeon’s decision. The fourth patient suffered from extensive afferent loop ischemia due to obstruction and strangulation. The fifth patient presented with generalized peritonitis owing to duodenal ischemia and necrosis with unknown cause. The sixth patient presented with hemorrhagic shock because of a huge duodenal ulcer at the mesenteric site, failed to endoscopic hemostasis and angiographic embolization. All of the patients had surgical complications of varying severity, including one case of in-hospital mortality. One patient was discharged after EPD with multi-organ failure (grade 4 surgical complication); however, she had repeated admission afterwards and died 8 months after the operation. The patient was also designated as surgery-related mortality. The APACHE II score ranged from 6 to 19 among these patients, while the patient who suffered from in hospital mortality carried the highest score. Two of the patients had underlying malignancy (33%). All the parameters, including age, BMI, albumin level, and intraoperative blood loss, had a wide range of values.

**Table 1:** Demographic data of patients who underwent emergent pancreaticoduodenectomy for non-traumatic etiologies in our series

Sex/age/indication	Cancer	Procedure/blood loss (ml)	APACHE II score/BMI/Hb (g/dL)/albumin (g/dL)	Complication/grading	Death	Hospital stay (days)
M/70/internal bleeding after partial pancreatectomy	No	CW/1000	17/18.6/13.6/4.9	Bile leakage/2	No	109
M/49/bleeding duodenal lymphoma	Lymphoma	CW/20	16/20.1/11/2.2	P-duct leakage with Peritonitis/3	No	54
F/78/A-loop perforation after ERCP	GC	CW/400	12/21.3/9.4/3.3	MOF/4	Yes^a^	16
F/58/A-loop obstruction and perforation	No	CW/500	6/17.3/11.9/3.2	Retro-peritoneal infection/3	No	17
M/32/bowel ischemia	No	CW/2850	19/24.3/11.3/1.9	Septic shock with liver and renal failure/5	Yes	11
F/39/duodenal ulcer bleeding	No	CW/1000	17/19.5/4.5/2.2	Wound infection/2	No	40
53.5		750	16.5/19.8/11.2/2.7			28.5

*ERCP* endoscopic retrograde cholangiopancreaticography, *GC* gastric cancer, *CW* classic Whipple’s operation, *BMI* body mass index, *MOF* multi-organ failure

^a^The patient had been discharged and died of complications of MOF 8 months after initial EPD

Table 2 presented 31 EPD accounting for 1.84% of all 2984 cases who underwent EPD for non-traumatic etiologies from the literature and the present series [8, 11, 14–22]. The rate of EPD for non-traumatic etiologies is similar between the present study and the other 3 series, ranging from 0.3 to 3%.

**Table 2:** Results of cases from literature review and our series

Study year	Author	PD	EPD		EPD for non-trauma		Mortality rate
2001	Tuech	N/A			1	N/A	0%
2002	Z’graggen	417	4	1.0%	4	1.0%	25%
2004	Sakakima	N/A			1	N/A	0%
2006	Maeda	N/A			1	N/A	0%
2007	Stratigos	N/A			1	N/A	0%
2010	Standop	301	6	2.0%	6	2.0%	16.7%
2010	Hecker	N/A			1	N/A	0%
2010	Michael	N/A			1	N/A	0%
2013	Lupaşcu	N/A			1	N/A	0%
2014	Gulla	1166	10	0.9%	3	0.3%	0%
2015	Lissidini	169	5	3.0%	5	3.0%	40%
	Present study	931	10	1.1%	6	0.6%	33.3%
	Overall	2984			31		19.4%

*PD* pancreaticoduodenectomy, *EPD* emergent pancreaticoduodenectomy

Table 3 shows the propensity score of age and gender in the cohort receiving elective PD for our database shows ratio of 1:2. EPDs disclosed mortality rate of 19.4 versus 3.2% (*P* = 0.015) in non-EPD.

**Table 3:** The 1:2 propensity score matching comparison (age and gender) between EPD and elective PD relating to surgical mortality

	EPD	Elective PD	*P* value
	31	62	
Gender			
Male	19	37	*P* = 1.000
Female	12	25	
Age			
Mean (±S.D.)	56.7 (14.5)	57.2 (14.5)	*P* = 0.889
Median	58.0	58.0	
Range	32-85	29-86	
Surgical mortality			
Yes	6	2	*P* = 0.015
No	25	60	
%	19.4	3.2	

*EPD* emergent pancreatoduodenectomy, *PD* pancreatoduodenectomy

Table 4 shows the association factors contributing to surgical mortality of EPD, including age, gender, etiology, malignancy, and length of hospital stay. However, only patients suffering from perforation who received EPDs are the independent factor associating with surgical mortality rate of EPD, demonstrated by the multivariate logistic regression analysis. (perforation versus bleeding; odds ratio = 39.494, *P* = 0.031).

**Table 4:** Univariate and multivariate logistic regression analysis of risk factors related to surgical mortality of the new cohort comprising 31 patients

Variables		Univariate analysis			Multivariate analysis		
		OR	95% CI	*P* value	OR	95% CI	*P* value
Age	Year	1.028	0.964–1.097	0.401	1.013	0.907–1.132	0.817
Gender	Male vs. female	3.929	0.399–38.704	0.241	19.174	0.468–785.328	0.119
Etiology	Perforation vs. bleeding	10.625	1.059–106.573	0.045	39.494	1.395–1117.856	0.031
Cancer	Cancer vs. no cancer	2.545	0.391–16.550	0.328	5.375	0.183–157.541	0.329
Hospital stay	Day	0.990	0.962–1.019	0.501	0.979	0.936–1.023	0.338

## Discussion

First, it should be emphasized that although the morbidity and mortality rate of PD had decreased since the first description of PD in 1935 [2], surgical outcome was still dismal in experienced institutes for decades. The meticulous preoperative preparation for the most complex abdominal operations has improved its outcomes. In this report, we demonstrated that EPD for non-trauma is an uncommonly performed procedure with incidence ranging from 0.3 to 3% among elective PDs.

Secondly, as shown in our report, the overall complication rate and surgical mortality rate for EPD are 83.9 and 19.4%, respectively. The propensity score match further disclosed the significantly higher surgical mortality of EPDs when compared with elective PDs (19.4 versus 3.2%). The possible explanation for the higher morbidity and mortality of EPD is that emergent surgeries possess potential unpreparedness with respect to elective surgeries [23].

Thirdly, and most importantly, is that the report clarified the preoperative predictors for poor outcome of EPD. As we know, the indications for emergent visceral operations can be narrowed to two aspects: perforation and bleeding. Visceral perforation induces secondary peritonitis and results in sepsis [24], which compromises the patient’s preoperative condition more than mere bleeding. Previous reports focused on the impact of etiology (perforation or bleeding) on surgical outcomes of emergent gastrointestinal surgery. For emergent gastrectomy, the etiology had no association with surgical mortality [25, 26]. Similar to previous reports regarding complicated colonic diverticular disease, perforation is one of the predictors for surgical mortality in EPD [27]. In this study, we gathered available preoperative parameters along with etiology for EPD to clarify the factors that affect surgical outcomes. As demonstrated in the univariate and the multivariate logistic regression analysis, patients suffering from perforation undergoing EPDs had significantly higher association with surgical mortality than those suffering from bleeding. However, the result may reflect the biased selection of this cohort because there were more case reports of successful EPDs in managing patient with intractable bleeding, and only one of the seven case reports is related to perforation.

## Conclusions

This study disclosed that the rates of EPD for non-traumatic etiologies are low, ranging from 0.3 to 3% among elective PDs. EPDs have relatively higher surgical mortalities compared to well-prepared, elective PDs. Among EPDs, patients suffering from perforation might have a higher risk of surgical mortality than those suffering from bleeding when the operation is warranted.

## Abbreviations

EPD: Emergent pancreaticoduodenectomy
PD: Pancreaticoduodenectomy
